# Outage Performance Analysis and SWIPT Optimization in Energy-Harvesting Wireless Sensor Network Deploying NOMA †

**DOI:** 10.3390/s19030613

**Published:** 2019-02-01

**Authors:** Hoang-Sy Nguyen, Tran Thai Hoc Ly, Thanh-Sang Nguyen, Van Van Huynh, Thanh-Long Nguyen, Miroslav Voznak

**Affiliations:** 1 VSB-Technical University of Ostrava, 17. listopadu 15/2172, 708 33 Ostrava-Poruba, Czech Republic; nhsy@bdu.edu.vn (H.-S.N.); ltthoc@ntt.edu.vn (T.T.H.L.); ntsang@bdu.edu.vn (T.-S.N.); longnt@hufi.edu.vn (T.-L.N.); miroslav.voznak@vsb.cz (M.V.); 2 Institute of Artificial Intelligence, Faculty of Information Technology, Binh Duong University, Thu Dau Mot City, Vietnam; 3 Nguyen Tat Thanh University, Ho Chi Minh City, Vietnam; 4 Modeling Evolutionary Algorithms Simulation and Artificial Intelligence, Faculty of Electrical & Electronics Engineering, Ton Duc Thang University, Ho Chi Minh City, Vietnam; 5 Center for Information Technology, Ho Chi Minh City University of Food Industry, Ho Chi Minh City, Vietnam

**Keywords:** wireless sensor network, energy-harvesting, NOMA, time-switching, power-splitting, outage probability, throughput

## Abstract

Thanks to the benefits of non-orthogonal multiple access (NOMA) in wireless communications, we evaluate a wireless sensor network deploying NOMA (WSN-NOMA), where the destination can receive two data symbols in a whole transmission process with two time slots. In this work, two relaying protocols, so-called time-switching-based relaying WSN-NOMA (TSR WSN-NOMA) and power-splitting-based relaying WSN-NOMA (PSR WSN-NOMA) are deployed to study energy-harvesting (EH). Regarding the system performance analysis, we obtain the closed-form expressions for the exact and approximate outage probability (OP) in both protocols, and the delay-limited throughput is also evaluated. We then compare the two protocols theoretically, and two optimization problems are formulated to reduce the impact of OP and optimize the data rate. Our numerical and simulation results are provided to prove the theoretical and analytical analysis. Thanks to these results, a great performance gain can be achieved for both TSR WSN-NOMA and PSR WSN-NOMA if optimal values of TS and PS ratios are found. In addition, the optimized TSR WSN-NOMA outperforms that of PSR WSN-NOMA in terms of OP.

## 1. Introduction

In recent years, fifth-generation (5G) wireless networks have acquired the reputation for improving energy efficiency (EE) more efficiently compared to conventional wireless networks [1,2]. Nevertheless, it is predicted that the growing number of mobile devices will pose threats to future wireless networks, i.e., wireless body area networks and wireless sensor networks (WSNs) [3]. This will lead to the rise in energy consumption of wireless sensor nodes. For example, if there are a massive number of Internet of Things (IoTs) sensor nodes or devices, battery replacement will not be ideal [4]. This motivates the search for green communications to improve EE [5,6,7,8]. Fortunately, thanks to energy-harvesting (EH) technologies, information transmission (IT), energy transfer, and the overall EE in low-power wireless networks can be boosted [9,10]. In principle, vibration, solar, wind, and geothermal are among popular sources of energy for EH which sometimes interrupt the process of EH due to their inconsistent availability. Therefore, EH using radio-frequency (RF) signals, which has increasingly attracted more research interest, is considered to be a promising source of energy. Besides that, simultaneous wireless information and power transfer (SWIPT), which has emerged as a potential technique, consists of two primary receiver architectures, i.e., power-splitting (PS) and time-switching (TS) [11,12]. Based on these two novel protocols, [13] produced two architectures based on PS and TS, so-called time-switching-based relaying (TSR) protocol and power-splitting-based relaying (PSR) protocol to make EH and information-processing possible at the relay node. The study in [14] took a half-duplex (HD) decode-and-forward (DF) small cell cognitive relay network (CRN) into consideration, where two TS-based policies were proposed so-called Optimal Time for Transmit Power at Source and Optimal Time for Transmit Power at Relay to maximize the transmit power at source and relay. In [15], PS protocol was deployed to study relay selection schemes, i.e., partial relay selection (PRS) scheme and optimal relay selection (ORS) scheme, where the authors comprehensively studied the outage probability (OP). In addition, the work in [16] obtained asymptotic closed-form expressions of OP and throughput over Rayleigh fading channel in cooperative CRN.

To meet the rising demand for green communications, non-orthogonal multiple access (NOMA) has been regarded as a prime technology for future wireless networks to boost the spectral efficiency (SE) [17,18,19,20]. In [17], a cooperative relaying system using NOMA was designed to enhance the SE. In this work, the average achievable rate was analyzed together with its asymptotic expression, and a suboptimal power allocation scheme for NOMA used at the source was proposed. In practice, signals of multiple NOMA users are superimposed at the transmitter, and a technique so-called successive interference cancellation (SIC) is applied at the receiver side to combine signals [18]. It is worth noting that a NOMA user with low channel gain is provided with high power, and vice versa. As a result, the NOMA user with better channel gain can decode information using SIC [19]. However, it does not apply for the situation when there is a NOMA user with low channel gain. Alternatively, the NOMA user with low channel gain decodes its information by treating high gain user’s signal as noise [20].

Due to the undeniable advantages of NOMA, deploying NOMA in different paradigms and applications has caught the attention of many researchers [21,22,23,24,25,26,27,28,29,30,31,32,33,34,35]. In [30], SWIPT was used in NOMA networks in which nodes are located randomly. Because the locations of users have a significant impact on the performance, three user selection schemes based on the user distances from the base station were proposed and compared, i.e., random near user and random far user (RNRF) selection, nearest near user and nearest far user (NNNF) selection, and nearest near user and farthest far user (NNFF) selection. Additionally, a NOMA cooperative relaying network was considered in [31], where the authors evaluated the system performance over Rician fading channels and obtained the exact expression for the average achievable rate. In [32], in the presence of self-interference, the achievable OP and the ergodic sum rate were studied, and the exact analytical expressions were obtained. In addition, the literature in [33] studied OP under the impact of the channel state information (CSI).

In [34,35], the authors focused on amplify-and-forward (AF) NOMA-based relaying networks. In these works, expressions for the exact and simple bounds of OP were obtained. Unlike the two aforementioned works, this paper presents the concept of SWIPT, in which we not only try to derive the exact and appropriate expressions for the OP but also consider and compare TSR and PSR protocols. Motivated by those aforementioned works, we try to point out the impact of TSR and PSR protocols on the system performance, and the ratios for those two architectures are also optimized to achieve the optimal data rate and better outage performance. Our primary contributions of this work are listed as follows:

- We obtain the closed-form expressions for the exact and approximate OP when TSR and PSR are deployed. Following that, we also provide the evaluation of the delay-limited throughput.
- To explore the system performance limits of the two receiver architectures, we compare them theoretically in terms of different values of TS and PS ratios. Further to this, we then work on two optimization problems to optimize the outage performance for TSR and PSR and the system data rates.
- Regarding the benefits of NOMA, we compare the traditional orthogonal multiple access (OMA) with our considered system in terms of OP and the achievable data rate. We prove the theoretical comparison between TSR and PSR via numerical results. Finally, we give a fair comparison with an existing cooperative relaying system using NOMA (CRS-NOMA) in [17] and a special comparison for OP in TSR WSN-NOMA, PSR WSN-NOMA, and RNRF selection for the far users [30].

We organize this paper as follows: The system model is presented in Section 2. The system performance for TSR WSN-NOMA and PSR WSN-NOMA is respectively discussed in Section 3 and Section 4 by obtaining closed-form expressions for the exact and approximate OP, and the delay-limited throughput is also studied. Section 5 gives the theoretical comparisons between the two protocols. Numerical results are presented in Section 6, which is followed by conclusions in Section 7.

## 2. System Model and Protocols

### 2.1. Network Model

**Figure 1:**
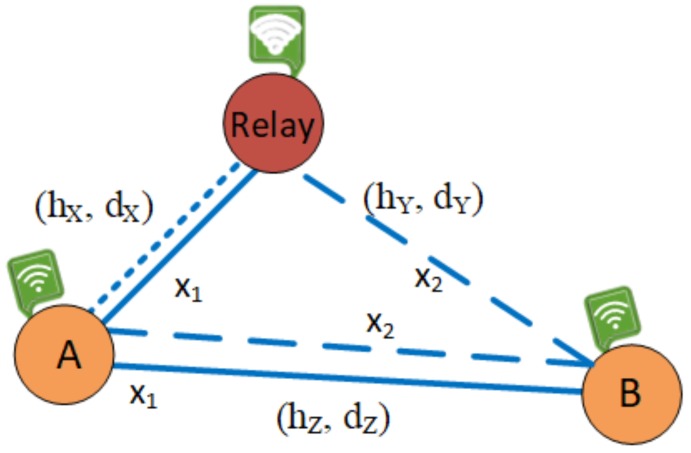
System model of a WSN-NOMA. The solid lines and the dash lines respectively represent the data transmission in the first time slot and the second time slot. Meanwhile, the half-dash lines stand for the energy transfer in both time slots [21].

In Figure 1, we study a wireless sensor network deploying non-orthogonal multiple access (WSN-NOMA), where there is a communication between a transmitter (A) and a receiver (B) via a relay (R). It is noted that R assists the communication between the two nodes due to the far distance and operates in HD DF relaying scheme. Thanks to the deployment of NOMA, the direct transmission between A and B can be carried out. It is assumed that the additive white Gaussian noise affects the received signal with zero mean $n_{0}$ and variance $N_{0}$. Furthermore, we respectively denote the distances between A–R, R–B, and A–B as $d_{X}$, $d_{Y}$, and $d_{Z}$, respectively. Besides that, the path-loss exponent is *m*.

It is noted that all nodes are equipped with a single antenna. An energy-constrained relay model can be implemented in different wireless systems, e.g., WSNs in toxic environments and wireless body area networks where sensors can be implanted inside the human body.

In addition, R exchanges a big amount of data with a limited rechargeable battery buffer, meaning that the total harvested energy at R must be used for IT.

Let us go through the system channels, there are two time slots involved in the transmission process. A transfers a data symbol, $x_{1}$ to R in the first time slot, i.e., $E\left\{{\left|x_{1}\right|}^{2}\right\}=1$ with the transmit power, $P_{A}$. A also transmits another data symbol denoted as $x_{2}$ to B in the second time slot with $P_{A}$ defined as $E\left\{{\left|x_{2}\right|}^{2}\right\}=1$ while B receives the data symbol, $x_{1}$ from R with the transmit power denoted as $P_{R}$. It is noted that power allocation at A is used to distinguish the two signal symbols thanks to their different characteristics in terms of receiving information. Besides, $h_{X}$, $h_{Y}$, and $h_{Z}$ respectively denoted as the channel coefficients of the links A–R, R–B, and A–B suffer from Rayleigh fading, in which the channel power gains denoted as ${\left|W\right|}^{2}$ with $W\in \left\{h_{X},h_{Y},h_{Z}\right\}$ are exponential distributed with mean value $\Omega_{W}$. For simplicity, as the fading gains of all links follow the Rayleigh distribution with the probability density function (PDF) which can be defined as

(1) $$f_{W}\left(x\right)=\frac{1}{\Omega_{W}}e^{-\frac{x}{\Omega_{W}}},$$

and we also express the cumulative distribution function (CDF) as

(2) $$F_{W}\left(x\right)=1-\frac{1}{\Omega_{W}}e^{-\frac{x}{\Omega_{W}}}.$$

Following from the considered model, we are going to present two protocols to comprehensively study the impact of EH on the system performance, i.e., TSR WSN-NOMA and PSR WSN-NOMA for this system in the following two subsections.

### 2.2. TSR WSN-NOMA Protocol

**Figure 2:**
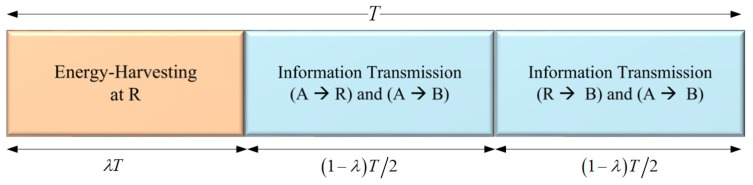
The general framework of TSR WSN-NOMA.

In Figure 2, the framework of TSR WSN-NOMA is depicted, where we take advantage of TS protocol with λ∈(0,1) being the TS ratio. In particular, the whole block time is denoted as *T*, in which (λT) is the harvested energy at R. In details, IT accounts for (T−λT), where half of that (T−λT)/2 is used for the A–R link while the R–B link makes up the remaining. Thus, the harvested energy, $E_{h}$ at R over a block time is defined as $E_{h}=\eta P_{A}d_{X}^{-m}{\left|X\right|}^{2}\lambda T$, where the energy conversion efficiency is 0<η<1 which relies on the rectification process and the EH circuitry.

**Figure 3:**
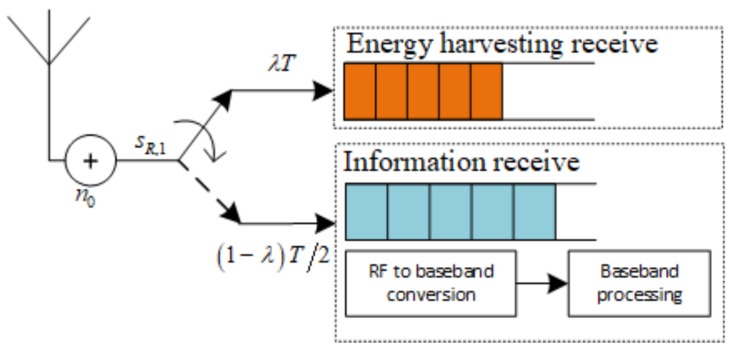
The model of R in case of TSR WSN-NOMA Protocol.

Following that we continue presenting TSR WSN-NOMA protocol in Figure 3, where $s_{R,1}$ representing the received RF signal at R is input into the EH receiver during λT subphase, and then input into to the information receiver for information decoding during the (1−λ)T subphase.

### 2.3. PSR WSN-NOMA Protocol

**Figure 4:**
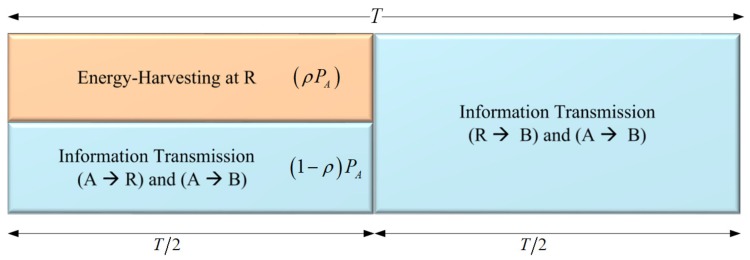
The general framework of a PSR WSN-NOMA.

In Figure 4, we present PSR WSN-NOMA protocol, where *T* is split evenly for the A–R and R–B links. During the first half, R devotes a part of the received signal power, $\rho P_{A}$ for the energy harvester while IT accounts for the remaining portion, $\left(1-\rho \right)P_{A}$, where 0≤ρ≤1 is the PS ratio.

**Figure 5:**
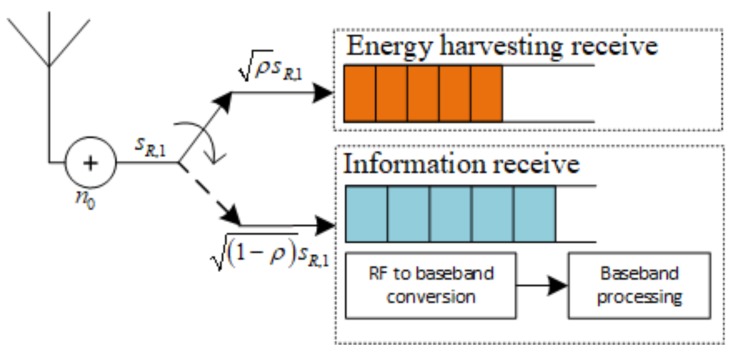
The model of R in case of PSR WSN-NOMA Protocol.

Likewise, we present the receiver model of for this protocol in Figure 5, where a power splitter divides $s_{R,1}$ into two parts for information-processing and EH in the proportion of ρ:1−ρ. Therefore, a part of the received signal $\sqrt{\rho }s_{R,1}$ is given to the EH receiver while the remaining portion, $\sqrt{\left(1-\rho \right)}s_{R,1}$ is allocated for the information receiver.

For simplicity, we summarize all the notations used in Table 1.

## 3. Performance Analysis for TSR WSN-NOMA

In this Section, we present the expressions for the exact and approximate OP, and the throughput in the delay-limited transmission mode is also investigated for our considered TSR protocol. First, we need to start with the evaluation of the transmission process in the first and second time slot.

### 3.1. The Transmission Process in the First Time Slot

To begin with, the signal symbol, $x_{1}$ received at R and B is respectively expressed as

(3) $$s_{R,1}=\sqrt{P_{A}d_{X}^{-m}}h_{X}x_{1}+n_{0},$$

and

(4) $$s_{B,1}=\sqrt{P_{A}d_{Z}^{-m}}h_{Z}x_{1}+n_{0}.$$

Hence, thanks to Equations (3) and (4), the received signal-to-noise ratios (SNRs) for data symbol, $x_{1}$ at R and B can be defined as

(5) $$\gamma_{R,1,TS}^{\left(x_{1}\right)}=\beta {\left|X\right|}^{2}d_{X}^{-m},$$

and

(6) $$\gamma_{B,1,TS}^{\left(x_{1}\right)}=\beta {\left|Z\right|}^{2}d_{Z}^{-m},$$

where the transmit SNR at A is $\beta =\frac{P_{A}}{N_{0}}$.

The decoded signal is transmitted from R with $E_{h}$ during (T−λT)/2, so $P_{R}$ can be written as

(7) $$P_{R,TS}=\frac{2E_{h}}{\left(T-\lambda T\right)}=2\eta \lambda {\left(1-\lambda \right)}^{-1}P_{A}{\left|X\right|}^{2}d_{X}^{-m}.$$

### 3.2. The Transmission Process in the Second Time Slot

Similarly, we compute the received signal at B as

(8) $$s_{B,2}=\sqrt{P_{R,TS}d_{Y}^{-m}}h_{Y}x_{1}+\sqrt{P_{A}d_{Z}^{-m}}h_{Z}x_{2}+n_{0}.$$

The above expression can be rewritten after substituting Equation (7) into Equation (8) as

(9) $$s_{B,2}=\sqrt{2\eta \lambda {\left(1-\lambda \right)}^{-1}P_{A}{\left|X\right|}^{2}d_{X}^{-m}d_{Y}^{-m}}h_{Y}x_{1}+\sqrt{P_{A}d_{Z}^{-m}}h_{Z}x_{2}+n_{0}.$$

In principle, because of the placement of nodes, the fading gain of the R–B link, $h_{Y}$ is bigger than that of the A–B link, $h_{Z}$. Due to the natural characteristics of different transceivers’ channels, it motivates us to apply NOMA in the second time slot. By taking advantage of the successive interference cancellation (SIC)-based NOMA scheme, B treats data symbol $x_{1}$ and $x_{2}$ as a noise term. To decode $x_{2}$, $x_{1}$ is mitigated from $s_{B,2}$. Hence, different from other NOMA systems [29], the received SNRs at B can be computed as

(10) $$\gamma_{B,2,TS}^{\left(x_{1}\right)}=\frac{2\eta \lambda {\left(1-\lambda \right)}^{-1}\beta d_{X}^{-m}d_{Y}^{-m}{\left|X\right|}^{2}{\left|Y\right|}^{2}}{\beta d_{Z}^{-m}{\left|Z\right|}^{2}+1},$$

and

(11) $$\gamma_{B,2,TS}^{\left(x_{2}\right)}=\beta {\left|Z\right|}^{2}d_{Z}^{-m}.$$

Due to the deployment of fixed DF scheme at R, the end-to-end SNR for data symbol $x_{1}$ can be expressed as

(12) $$\gamma_{e2e,TS}^{\left(x_{1}\right)}=\min\left\{\gamma_{R,1,TS}^{\left(x_{1}\right)},\gamma_{B,2,TS}^{\left(x_{1}\right)}\right\}.$$

### 3.3. Outage Performance

#### 3.3.1. Exact Expression of the Outage Probability

In principle, OP is presented as the probability, where the instantaneous SNR, γ is set below the pre-defined threshold, $\gamma_{0}$. For simplicity, the OP is defined as $OP=Pr\left(\gamma <\gamma_{0}\right)=F_{\gamma }\left(\gamma_{0}\right)$.

**Proposition** **1.** *The OPs for data symbols, $x_{1}$ and $x_{2}$ using NOMA can be respectively expressed as*

(13) $$OP_{TS}^{\left(x_{1}\right)}=1-e^{-\varepsilon_{1}}-e^{-\varepsilon_{2a}}\left(1-e^{-\varepsilon_{1}}\right)\int_{z=0}^{\infty }\frac{1}{\Omega_{Z}}e^{-\frac{z}{\Omega_{Z}}}\Theta_{TS}K_{1}\left(\Theta_{TS}\right)dz,$$

*and*

(14) $$OP_{TS}^{\left(x_{2}\right)}=1-e^{-\varepsilon_{1}}\int_{z=0}^{\infty }\frac{1}{\Omega_{Z}}e^{-\frac{z}{\Omega_{Z}}}\Theta_{TS}K_{1}\left(\Theta_{TS}\right)dz,$$

*where $\varepsilon_{1}=\frac{d_{Z}^{m}\gamma_{0}}{\beta \Omega_{Z}}$, $\varepsilon_{2a}=\frac{d_{X}^{m}\gamma_{0}}{\beta \Omega_{X}}$, and $\Theta_{TS}=\sqrt{\frac{4d_{X}^{m}d_{Y}^{m}\gamma_{0}\left(\beta d_{Z}^{-m}z+1\right)}{2\eta \lambda {\left(1-\lambda \right)}^{-1}\beta \Omega_{X}\Omega_{Y}}}$.*

**Proof.**  The following expressions are obtained because of the derived CDF functions for $\gamma_{R,1,TS}^{\left(x_{1}\right)}$ and $\gamma_{B,1,TS}^{\left(x_{1}\right)}$ as

(15) $$F_{\gamma_{R,1,TS}^{\left(x_{1}\right)}}\left(\gamma_{0}\right)=1-e^{-\frac{d_{X}^{m}\gamma_{0}}{\beta \Omega_{X}}},$$

and

(16) $$F_{\gamma_{B,1,TS}^{\left(x_{1}\right)}}\left(\gamma_{0}\right)=F_{\gamma_{B,2,TS}^{\left(x_{2}\right)}}\left(\gamma_{0}\right)=1-e^{-\frac{d_{Z}^{m}\gamma_{0}}{\beta \Omega_{Z}}}.$$

We need to consider the CDF of $\gamma_{B,2,TS}^{\left(x_{1}\right)}$ first before the OP for $x_{1}$ can be computed. Therefore, the CDF function of $F_{\gamma_{B,2,TS}^{\left(x_{1}\right)}}\left(\gamma_{0}\right)$ is conditioned on ${\left|Z\right|}^{2}$ by

(17) $$\begin{array}{c} F_{\gamma_{B,2,TS}^{\left(x_{1}\right)}}\left(\gamma_{0}\right)=\mathrm{Pr}\left({\left|X\right|}^{2}\leq \frac{\gamma_{0}d_{X}^{m}d_{Y}^{m}\left(\beta {\left|Z\right|}^{2}d_{Z}^{-m}+1\right)}{2\eta \lambda {\left(1-\lambda \right)}^{-1}\beta {\left|Y\right|}^{2}}\right)\\ \;\;=\frac{1}{\Omega_{Y}}\int_{0}^{\infty }\left(1-e^{-\frac{1}{y}\left(\frac{\gamma_{0}d_{X}^{m}d_{Y}^{m}\left(\beta d_{Z}^{-m}{\left|Z\right|}^{2}+1\right)}{2\eta \lambda {\left(1-\lambda \right)}^{-1}\beta \Omega_{X}}\right)}\right)e^{-\frac{y}{\Omega_{Y}}}dy\\ \;\;=1-K_{1}\left(\sqrt{\frac{4d_{X}^{m}d_{Y}^{m}\gamma_{0}\left(\beta d_{Z}^{-m}{\left|Z\right|}^{2}+1\right)}{2\eta \lambda {\left(1-\lambda \right)}^{-1}\beta \Omega_{X}\Omega_{Y}}}\right)\times \sqrt{\frac{4d_{X}^{m}d_{Y}^{m}\gamma_{0}\left(\beta d_{Z}^{-m}{\left|Z\right|}^{2}+1\right)}{2\eta \lambda {\left(1-\lambda \right)}^{-1}\beta \Omega_{X}\Omega_{Y}}} \end{array},$$

where we derive the last formula by using $\int_{0}^{\infty }e^{-\frac{\beta }{4x}-\alpha x}dx=\sqrt{\frac{\beta }{\alpha }}K_{1}\left(\sqrt{\beta \alpha }\right)$, ([36], Equation (3.324.1)). Thus, the CDF of $\gamma_{B,2,TS}^{\left(x_{1}\right)}$ is rewritten over ${\left|Z\right|}^{2}$ by

(18) $$F_{\gamma_{B,2,TS}^{\left(x_{1}\right)}}\left(\gamma_{0}\right)=1-\frac{1}{\Omega_{Z}}\int_{0}^{\infty }\left(e^{-\frac{z}{\Omega_{Z}}}\Theta_{TS}K_{1}\left(\Theta_{TS}\right)\right)dz.$$

If the data symbol, $x_{1}$ is decoded, there will be an outage event. Thanks to Equations (15) and (18), we derive the end-to-end SNR OP at B as

(19) $$\begin{array}{c} OP_{e2e,TS}^{\left(x_{1}\right)}=\mathrm{Pr}\left(\min\left\{\gamma_{R,1,TS}^{\left(x_{1}\right)},\gamma_{B,2,TS}^{\left(x_{1}\right)}\right\}\leq \gamma_{0}\right)\\ \;\;=1-\left(1-F_{\gamma_{R,1,TS}^{\left(x_{1}\right)}}\left(\gamma_{0}\right)\right)\times \left(1-F_{\gamma_{B,2,TS}^{\left(x_{1}\right)}}\left(\gamma_{0}\right)\right)\\ \;\;=1-\frac{1}{\Omega_{Z}}\int_{0}^{\infty }\left(e^{-\frac{z}{\Omega_{Z}}-\frac{d_{X}^{m}\gamma_{0}}{\beta \Omega_{X}}}\Phi_{TS}K_{1}\left(\Phi_{TS}\right)\right)dz. \end{array}$$

Thanks to selection combining technique applied at R, the total expression for $x_{1}$ is

(20) $$OP_{TS}^{\left(x_{1}\right)}=F_{\gamma_{B,1,TS}^{\left(x_{1}\right)}}\left(\gamma_{0}\right)\times OP_{e2e,TS}^{\left(x_{1}\right)}.$$

Substituting Equations (16) and (19) into Equation (20), (13) can be derived.

Additionally, the OP for $x_{2}$ for the A–B link, $OP_{TS}^{\left(x_{2}\right)}$ can be computed by

(21) $$OP_{TS}^{\left(x_{2}\right)}=1-\left(1-F_{\gamma_{B,2,TS}^{\left(x_{1}\right)}}\left(\gamma_{0}\right)\right)\times \left(1-F_{\gamma_{B,2,TS}^{\left(x_{2}\right)}}\left(\gamma_{0}\right)\right),$$

where $F_{\gamma_{B,2,TS}^{\left(x_{2}\right)}}\left(\gamma_{0}\right)$ is presented in Equation (16).

This ends the proof for Proposition 1. ☐

#### 3.3.2. Approximate Expressions of the Outage Probability

Due to the difficulty in deriving closed-form expressions for OP with theoretical analysis in Equations (13) and (14) as shown Proposition 1, we are going to obtain the approximate expressions for OP at extremely high-SNR regime in the following Proposition 2.

**Proposition** **2.** *As a result, it is relatively easy to obtain the approximate result of Proposition 1 at high SNR which can be expressed by*

(22) $$OP_{TS,\infty }^{\left(x_{1}\right)}\approx \varepsilon_{1}-\varepsilon_{1}\left(1-\varepsilon_{2a}\right)e^{\frac{1}{2}\Theta_{TS,\infty }}W_{-1,\frac{1}{2}}\left(\Theta_{TS,\infty }\right),$$

*and*

(23) $$OP_{TS,\infty }^{\left(x_{2}\right)}\approx 1-\left(1-\varepsilon_{1}\right)e^{\frac{1}{2}\Theta_{TS,\infty }}W_{-1,\frac{1}{2}}\left(\Theta_{TS,\infty }\right),$$

*where $\Theta_{TS,\infty }=\frac{1}{2}\frac{d_{X}^{m}d_{Y}^{m}d_{Z}^{-m}\Omega_{Z}\gamma_{0}}{\eta \lambda {\left(1-\lambda \right)}^{-1}\Omega_{X}\Omega_{Y}}$.*

**Proof.**  Following the similar steps in the proof for Proposition 1, we can upper bound the modified Bessel function of the second kind as, $xK_{1}\left(x\right)\to 1$, when x→0. Thus, in case of high SNR, β→∞, the CDF in Equation (18) is rewritten as

(24) $$\begin{array}{c} F_{\gamma_{B,2,TS}^{\left(x_{1}\right)}}\left(\gamma_{0}\right)=1-\int_{z=0}^{\infty }e^{-\frac{z}{\Omega_{Z}}}\sqrt{\frac{4d_{X}^{m}d_{Y}^{m}\gamma_{0}\left(\beta d_{Z}^{-m}z+1\right)}{2\eta \lambda {\left(1-\lambda \right)}^{-1}\beta \Omega_{X}\Omega_{Y}}}K_{1}\left(\sqrt{\frac{4d_{X}^{m}d_{Y}^{m}\gamma_{0}\left(\beta d_{Z}^{-m}z+1\right)}{2\eta \lambda {\left(1-\lambda \right)}^{-1}\beta \Omega_{X}\Omega_{Y}}}\right)dz\\ \;\;\;\leq 1-\frac{1}{\Omega_{Z}}\int_{z=0}^{\infty }e^{-\frac{z}{\Omega_{Z}}}\sqrt{\frac{4d_{X}^{m}d_{Y}^{m}d_{Z}^{-m}\gamma_{0}z}{2\eta \lambda {\left(1-\lambda \right)}^{-1}\Omega_{X}\Omega_{Y}}}K_{1}\left(\sqrt{\frac{4d_{X}^{m}d_{Y}^{m}d_{Z}^{-m}\gamma_{0}z}{2\eta \lambda {\left(1-\lambda \right)}^{-1}\Omega_{X}\Omega_{Y}}}\right)dz \end{array}.$$

Then, thanks to the integral identity $\int_{0}^{\infty }x^{\mu -\frac{1}{2}}e^{-\alpha x}K_{2v}\left(2\beta \sqrt{x}\right)dx=\frac{\Gamma \left(\mu +v+\frac{1}{2}\right)\Gamma \left(\mu -v+\frac{1}{2}\right)}{2\beta }e^{\frac{\beta^{2}}{2\alpha }}\alpha^{-\mu }W_{-\mu ,v}\left(\frac{\beta^{2}}{\alpha }\right)$ in ([36], Equation (6.643.2)), we derive

(25) $$F_{\gamma_{B,2,TS}^{\left(x_{1}\right)}}\left(\gamma_{0}\right)\leq 1-e^{\frac{1}{2}\frac{d_{X}^{m}d_{Y}^{m}d_{Z}^{-m}\Omega_{Z}\gamma_{0}}{2\eta \lambda {\left(1-\lambda \right)}^{-1}\Omega_{X}\Omega_{Y}}}W_{-1,\frac{1}{2}}\left(\frac{d_{X}^{m}d_{Y}^{m}d_{Z}^{-m}\Omega_{Z}\gamma_{0}}{2\eta \lambda {\left(1-\lambda \right)}^{-1}\Omega_{X}\Omega_{Y}}\right).$$

Finally, we can apply the approximations of $1-e^{-x}=x$ when x→0 in ([36], Equation (1.211.1)) on Equation (13). After some algebraic manipulations, Equation (22) can be obtained to finish the proof for Proposition 2. ☐

### 3.4. Throughput Performance

Given that the transmitter is communicating $R_{0}$ (bps/Hz) and (1−λ)T/2 is the effective communication time from A to B via R during *T*. Thus, the throughput in the delay-limited transmission mode, $\tau_{TS}^{\left(x_{1}/x_{2}\right)}$ is given by

(26) $$\tau_{TS}^{\left(x_{1}/x_{2}\right)}=\frac{\left(1-\lambda \right)}{2}\left(1-OP_{TS}^{\left(x_{1}/x_{2}\right)}\right)R_{0},$$

where the OP, $OP_{TS}^{\left(x_{1}/x_{2}\right)}$ can be calculated using Propositions 1 and 2.

## 4. Performance Analysis for PSR WSN-NOMA

Similar to what we have done the previous section for TSR WSN-NOMA, we are going to evaluate the impact of PSR on the system performance with expressions for the exact and approximate OP and delay-limited throughput. Let us start with the transmission process in the first and second time slot.

### 4.1. The Transmission Process in the First Time Slot

As illustrated in Figure 4, the received signal at R can be expressed in the first phase as

(27) $$\sqrt{\rho }s_{R,1}=\sqrt{\rho P_{A}d_{X}^{-m}}h_{X}x_{1}+n_{0}.$$

Following from Equation (27), the harvested energy at R is computed as

(28) $$E_{h}=\frac{1}{2}\eta \rho P_{A}d_{X}^{-m}{\left|X\right|}^{2}T.$$

Because we denote $E_{h}$ as the source power during T/2, $P_{R,PS}$ can be expressed as

(29) $$P_{R,PS}=\frac{2E_{h}}{T}=\eta \rho P_{A}{\left|X\right|}^{2}d_{X}^{-m}.$$

Nevertheless, the data symbol, $x_{1}$ received at R is calculated as

(30) $$\sqrt{1-\rho }s_{R,1}=\sqrt{\left(1-\rho \right)P_{A}d_{X}^{-m}}h_{X}x_{1}+n_{0}.$$

Similar to TSR WSN-NOMA, the same data is transmitted to B in the first time slot, so the SNR for $x_{1}$ received at B, $\gamma_{B,1,PS}^{\left(x_{1}\right)}$ is derived as in Equation (4).

Therefore, based on Equation (30), the SNR for $x_{1}$ at R is written as

(31) $$\gamma_{R,1,PS}^{\left(x_{1}\right)}=\beta \left(1-\rho \right){\left|X\right|}^{2}d_{X}^{-m}.$$

### 4.2. The Transmission Process in the Second Time Slot

It is noted that DF scheme at R first decodes the signal in Equation (30), re-modulates and finally forwards it with the harvested energy in Equation (29). Interestingly, B can deploy SIC to decode data transmitted from A successfully. Hence, the received signal at B, in this case, is written as

(32) $$s_{B,2}=\sqrt{P_{R,PS}d_{Y}^{-m}}h_{Y}x_{1}+\sqrt{P_{A}d_{Z}^{-m}}h_{Z}x_{2}+n_{0}.$$

Substituting Equation (29) into Equation (32), (32) is rewritten as

(33) $$s_{B,2}=\sqrt{\eta \rho P_{A}d_{X}^{-m}d_{Y}^{-m}}h_{X}h_{Y}x_{1}+\sqrt{P_{A}d_{Z}^{-m}}h_{Z}x_{2}+n_{0}.$$

Following that, the received SNRs at B for $x_{1}$ can be computed as

(34) $$\gamma_{B,2,PS}^{\left(x_{1}\right)}=\frac{\eta \rho \beta d_{X}^{-m}d_{Y}^{-m}{\left|X\right|}^{2}{\left|Y\right|}^{2}}{\beta d_{Z}^{-m}{\left|Z\right|}^{2}+1}.$$

Similarly, we define the received SNRs at B for data symbol $x_{2}$, $\gamma_{B,2,PS}^{\left(x_{2}\right)}$ as in Equation (11).

Based on Equations (31) and (34), the end-to-end SNR for $x_{1}$ using PSR protocol is derived as

(35) $$\gamma_{e2e,PS}^{\left(x_{1}\right)}=\min\left\{\gamma_{R,1,PS}^{\left(x_{1}\right)},\gamma_{B,2,PS}^{\left(x_{1}\right)}\right\}.$$

### 4.3. Outage Performance

#### 4.3.1. Exact Expression of the Outage Probability

**Proposition** **3.** *For this case, the OP can be analytically obtained for data symbols, $x_{1}$ and $x_{2}$ at B as (We the specific steps of studying OP for the PSR WSN-NOMA omit here since most of the steps follow from the proof for Proposition 1.)*

(36) $$OP_{PS}^{\left(x_{1}\right)}=1-e^{-\varepsilon_{1}}-e^{-\varepsilon_{2b}}\left(1-e^{-\varepsilon_{1}}\right)\int_{z=0}^{\infty }\frac{1}{\Omega_{Z}}e^{-\frac{z}{\Omega_{Z}}}\Theta_{PS}K_{1}\left(\Theta_{PS}\right)dz,$$

*and*

(37) $$OP_{PS}^{\left(x_{2}\right)}=1-e^{-\varepsilon_{1}}\int_{z=0}^{\infty }\frac{1}{\Omega_{Z}}e^{-\frac{z}{\Omega_{Z}}}\Theta_{PS}K_{1}\left(\Theta_{PS}\right)dz,$$

*where $\varepsilon_{1}=\frac{d_{Z}^{m}\gamma_{0}}{\beta \Omega_{Z}}$, $\varepsilon_{2b}=\frac{d_{X}^{m}\gamma_{0}}{\beta \left(1-\rho \right)\Omega_{X}}$, and $\Theta_{PS}=\sqrt{\frac{4d_{X}^{m}d_{Y}^{m}\gamma_{0}}{\eta \rho \beta \Omega_{X}\Omega_{Y}}\left(\beta d_{Z}^{-m}z+1\right)}$.*

#### 4.3.2. Approximate Expressions of the Outage Probability

It is worth noting that the theoretical analysis of OP in Equations (36) and (37) is difficult to obtain closed-form expressions with traditional techniques due to requiring the modified Bessel functions. For the simplicity, we are going to derive the approximate expressions for OP at extremely high-SNR regime in the following Proposition 4.

**Proposition** **4.** *In this case, we analytically compute the approximate expressions of the OP can be as (We omit the detailed derivations of the OP for this case because we follow the similar steps taken in proof for Proposition 2.)*

(38) $$OP_{PS,\infty }^{\left(x_{1}\right)}\approx \varepsilon_{1}-\varepsilon_{1}\left(1-\varepsilon_{2b}\right)e^{\frac{1}{2}\Theta_{PS,\infty }}W_{-1,\frac{1}{2}}\left(\Theta_{PS,\infty }\right),$$

*and*

(39) $$OP_{PS,\infty }^{\left(x_{2}\right)}\approx 1-\left(1-\varepsilon_{1}\right)e^{\frac{1}{2}\Theta_{PS,\infty }}W_{-1,\frac{1}{2}}\left(\Theta_{PS,\infty }\right),$$

*where $\Phi_{PS,\infty }=\frac{d_{X}^{m}d_{Y}^{m}d_{Z}^{-m}\Omega_{Z}\gamma_{0}}{\eta \rho \Omega_{X}\Omega_{Y}}$.*

### 4.4. Throughput Performance

Due to being T/2 the effective communication time between A and B in *T*. The delay-limited throughput, $\tau_{PS}^{\left(x_{1},x_{2}\right)}$ considering PSR can be given by

(40) $$\tau_{PS}^{\left(x_{1}/x_{2}\right)}=\frac{1}{2}\left(1-OP_{PS}^{\left(x_{1}/x_{2}\right)}\right)R_{0},$$

where the OP, $OP_{PS}^{\left(x_{1}/x_{2}\right)}$ is expressed using Propositions 3 and 4.

**Remark** **1.** *For simplicity, we summarize the derived expressions for OP for both TSR WSN-NOMA and the PSR WSN-NOMA protocols in Table 2. It is easy to see that there are major changes in the OP as TS ratio and PS ratio λ, ρ vary between 0 and 1. Because of the rise in λ or ρ, there will be more transmit power at R. As a result, there will be fewer outage events. In addition, we are going to discuss the system OPs for TSR and PSR the following λ, ρ ratios in the following session.*

## 5. Theoretical Comparison and Optimal Problem of TSR WSN-NOMA and PSR WSN-NOMA

In this section, for further insights into TSR WSN-NOMA and PSR WSN-NOMA protocols, we are going to compare them theoretically with different values of λ and ρ. Besides that, we are also going to optimize λ and ρ ratios to optimize the data rates which accordingly contribute to fewer outage events. Now, let us start with the comparison between TSR and PSR.

### 5.1. Theoretical Comparison of TSR WSN-NOMA and PSR WSN-NOMA

In principle, the comparison between two systems requires us to define $P_{R,TS/PS}$ as in Equations (7) and (28) summarized in Table 2 with different values of λ and ρ ratios. Regarding the system OPs for both data symbols, we express $OP_{TS/PS}^{\left(x_{1}\right)}$ and $OP_{TS/PS}^{\left(x_{2}\right)}$ following from Equations (13), (14), (36), and (37) in terms of finding exact expressions while Equations (22), (23), (38) and (39) are used in case of high-SNR approximation. They are all expressed by variables, $\varepsilon_{1},\varepsilon_{2a},\varepsilon_{2b},\Theta_{TS}$ and $\Theta_{PS}$ which are different in TSR WSN-NOMA and PSR WSN-NOMA protocols as shown in Table 2.

#### 5.1.1. Case 1. λ=ρ

According to Table 2, it can be seen that $P_{R,TS}>P_{R,PS}$, and $\varepsilon_{2a}<\varepsilon_{2b}$ with 0<λ,ρ<1. The end-to-end SNR, $\gamma_{e2e}$ increases as A’s transmit power rises. Next, $\gamma_{R,1}\left(\gamma_{B,2}\right)$ is the monotonically decreasing (increasing) function of $P_{R}$. Therefore, $OP_{TS,\infty }^{\left(x_{1}\right)}<OP_{PS,\infty }^{\left(x_{1}\right)}$. As a result, the system OP for TSR WSN-NOMA is better than that of PSR WSN-NOMA in Case 1.

#### 5.1.2. Case 2. λ>ρ

We have $P_{R,TS}>P_{R,PS}$. Therefore, the system OP for PSR WSN-NOMA is superior to that of TSR WSN-NOMA in Case 2.

#### 5.1.3. Case 3. λ<ρ

In this case, it is uncertain to determine whether $P_{R,TS}>P_{R,PS}$ with λ<ρ. Motivated from this, $\varepsilon_{2a}>\varepsilon_{2b}$, we are going to compare them theoretically by providing numerical results.

### 5.2. Performance Optimization

For this part, we are going to evaluate the instantaneous capacities at R which rely on λ and ρ ratios and other parameters. The achievement of optimal λ and ρ ratios can greatly enhance the data rate which accordingly improves the reliability of this communication system. This also contributes to better EE, meaning that data rate achieves maximum within the given transmit power.

#### 5.2.1. Optimization Problem for TSR WSN-NOMA

First, we express the data transmission rate as

(41) $$R_{TS}^{\left(x_{1}\right)}\left(\lambda \right)=\left\{\frac{\left(1-\lambda \right)}{2}\log_{2}\left(1+\min\left(\gamma_{R,1,TS}^{\left(x_{1}\right)},\gamma_{B,2,TS}^{\left(x_{1}\right)}\right)\right)\right\}.$$

Following that, the following optimization must be solved before the optimal, $\lambda^{*}$ can be achieved as

(42) $$\lambda^{*}\underset{0<\lambda <1}{=}\arg\max_{\lambda }\left\{\frac{\left(1-\lambda \right)}{2}\log_{2}\left(1+\min\left(\gamma_{R,1,TS}^{\left(x_{1}\right)},\gamma_{B,2,TS}^{\left(x_{1}\right)}\right)\right)\right\}.$$

It is noted that the optimization problem above can be solved analytically, which is explained in detail in the following Proposition 5.

**Proposition** **5.** The average optimal TS ratio, $\lambda^{*}$ is expressed by

(43) $$\lambda^{*}=\left\{\begin{array}{c} \frac{e^{W\left(\frac{{\bar{\upsilon }}_{1}-1}{e}\right)+1}-1}{{\bar{\upsilon }}_{1}+e^{W\left(\frac{{\bar{\upsilon }}_{1}-1}{e}\right)+1}-1},\;\mathrm{if}\;e^{W\left(\frac{{\bar{\upsilon }}_{1}-1}{e}\right)+1}<{\bar{\upsilon }}_{1}+1\\ \frac{1}{{\bar{\upsilon }}_{2}+1},\;\mathrm{otherwise} \end{array}\right.,$$

*where ${\bar{\upsilon }}_{1}=\frac{2\eta \beta d_{X}^{-m}d_{Y}^{-m}\Omega_{X}\Omega_{Y}}{\beta d_{Z}^{-m}\Omega_{Z}+1}$, and ${\bar{\upsilon }}_{2}=\frac{\beta d_{Z}^{-m}\Omega_{Z}+1}{2\eta d_{Y}^{-m}\Omega_{Y}}$.*

**Proof.**  Following from Equations (5), (10), we define $\upsilon_{2}^{2}=\upsilon_{1}\upsilon_{3}$, where $\upsilon_{1}=\frac{2\eta \beta d_{X}^{-m}d_{Y}^{-m}{\left|X\right|}^{2}{\left|Y\right|}^{2}}{\beta d_{Z}^{-m}{\left|Z\right|}^{2}+1}$, $\upsilon_{3}\;=\;\beta {\left|X\right|}^{2}d_{X}^{-m}$. To this end, it is easy to study two separate regions.

Considering region i.) with $\lambda \in \left(0,{\left(1+\upsilon_{2}\right)}^{-1}\right)$, we decided to take the first derivative of the instantaneous data rate as $R_{TS}^{\left(x_{1}\right)}\left(\lambda \right)=\left\{\frac{\left(1-\lambda \right)}{2}\log_{2}\left(1+\lambda {\left(1-\lambda \right)}^{-1}\upsilon_{1}\right)\right\}$ with respect to λ and set $\frac{\delta R_{TS}^{\left(x_{1}\right)}\left(\lambda \right)}{\delta \lambda }=0$. Therefore, we have

(44) $$\upsilon_{1}+\lambda {\left(1-\lambda \right)}^{-1}\upsilon_{1}=\left(1+\lambda {\left(1-\lambda \right)}^{-1}\upsilon_{1}\right)\ln\left(1+\lambda {\left(1-\lambda \right)}^{-1}\upsilon_{1}\right).$$

After some algebraic manipulations, we can rewrite that expression as

(45) $$\ln\left(\frac{z}{e}\right)e^{\ln\left(\frac{z}{e}\right)}=\frac{\upsilon_{1}-1}{e},$$

where $z=1+\lambda {\left(1-\lambda \right)}^{-1}\upsilon_{1}$.

Thanks to the use of Lambert *W* function $\ln\left(\frac{z}{e}\right)=W\left(\frac{\upsilon_{1}-1}{e}\right)$, we can derive the desirable result as

(46) $$\lambda_{1}^{*}=\frac{e^{W\left(\frac{\upsilon_{1}-1}{e}\right)+1}-1}{\upsilon_{1}+e^{W\left(\frac{\upsilon_{1}-1}{e}\right)+1}-1}.$$

Turning into the second region ii.) With $\lambda \in \left[{\left(1+\upsilon_{2}\right)}^{-1},1\right)$, we take the first derivative, $R_{TS}^{\left(x_{1}\right)}\left(\lambda \right)$ with respect to λ which is a decreasing function to below zero. Thus, the optimal EH time is computed as

(47) $$\lambda_{2}^{*}={\left(1+\upsilon_{2}\right)}^{-1}$$

It is noticeable that if $\lambda_{1}^{*}\in \left(0,{\left(1+\upsilon_{2}\right)}^{-1}\right)$, there will be two scenarios, e.g., $\lambda_{1}^{*}$ is optimal or $\lambda_{2}^{*}$ is optimal.

This ends proof for Proposition 5. ☐

#### 5.2.2. Optimization Problem for PSR WSN-NOMA

Regarding PSR WSN-NOMA, we achieve the optimal $\lambda^{*}$ by solving the following optimization

(48) $$\rho^{*}\underset{0<\rho <1}{=}\arg\max_{\rho }\left\{\frac{1}{2}\log_{2}\left(1+\min\left(\gamma_{R,1,PS}^{\left(x_{1}\right)},\gamma_{B,2,PS}^{\left(x_{1}\right)}\right)\right)\right\}.$$

After we characterize the end-to-end SNR, we can simplify the optimal $\rho^{*}$ to

(49) $$\rho^{*}=\underset{0<\rho <1}{\arg\max\min}\left(\gamma_{R,1,PS}^{\left(x_{1}\right)},\gamma_{B,2,PS}^{\left(x_{1}\right)}\right).$$

The average optimal value $\rho^{*}$ is derived by solving the following equation, $\gamma_{R,1,PS}^{\left(x_{1}\right)}=\gamma_{B,2,PS}^{\left(x_{1}\right)}$. Thus, the desired result, $\rho^{*}$ can be obtained after some simple algebraic manipulations as

(50) $$\rho *={\left(\frac{\eta d_{Y}^{-m}\Omega_{Y}}{\beta d_{Z}^{-m}\Omega_{Z}+1}+1\right)}^{-1}.$$

## 6. Numerical Results

In this section, some simulation results in terms of the OP, throughput and the achievable data rate are provided, and we also compare them with complementary Monte Carlo-simulated performance results. The simulation model is developed in MATLAB for the overall evaluation of the considered system. For generality, $10^{6}$ realizations of Rayleigh distribution RVs are generated, and the following parameters are set as λ=ρ=0.2, η=80%, $R_{0}=1$ (dB), and m=3. In addition, the distances are set as $d_{Z}=2$, $d_{X}=1$, and $d_{Y}=d_{Z}-d_{X}$ while the mean value of the exponential RVs is set to 1.

**Figure 6:**
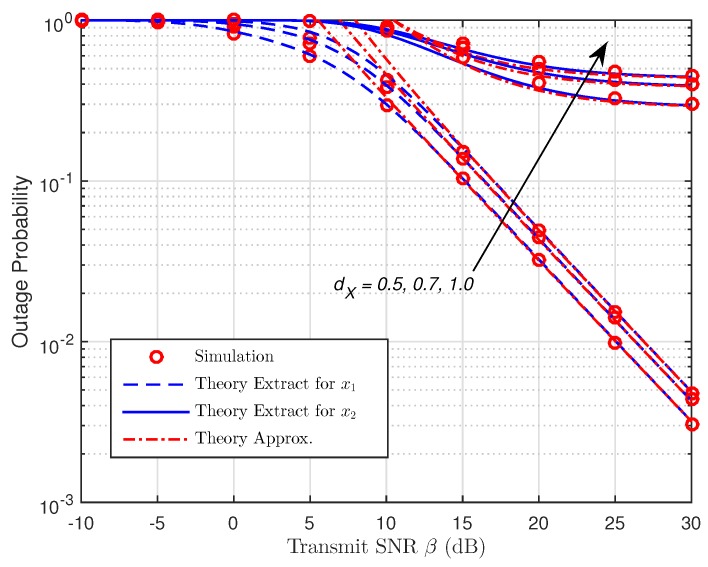
OP versus the transmit SNR (TSR WSN-NOMA).

**Figure 7:**
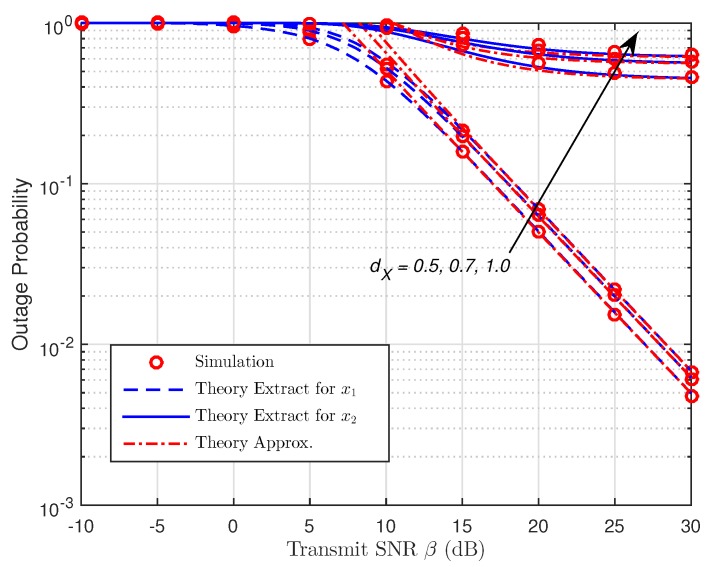
OP versus the transmit SNR (PSR WSN-NOMA).

As presented in Figure 6 and Figure 7, the OP is shown as a function of β with different placements of R for both protocols. We can spot that the OP rises as $d_{X}$ increases, so the shorter the distance of the R–B link is, the better the data rate becomes due to the assistance of R with NOMA. In addition, if there is an increase in $d_{X}$, the harvested energy and the signal receiving capacity at R will fall due to the larger path loss, $d_{X}^{-m}$. As a result, it leads to the poor signal reception strength at B which accordingly degrades the performance system. However, the gaps between the corresponding curves can be witnessed when β increases. The accuracy can be enhanced in case of small values of β. It is noted that the OP for data symbol, $x_{2}$ falls to a constant value due to the increase of β, meaning that the OP for $x_{2}$ is restricted by the distance between R and B. It is shown that the diversity orders of $x_{1}$ and $x_{2}$ are one, and they are estimated correctly. Furthermore, the high-SNR approximations are relatively tight at moderate SNRs, and they gradually become exact at high SNRs.

**Figure 8:**
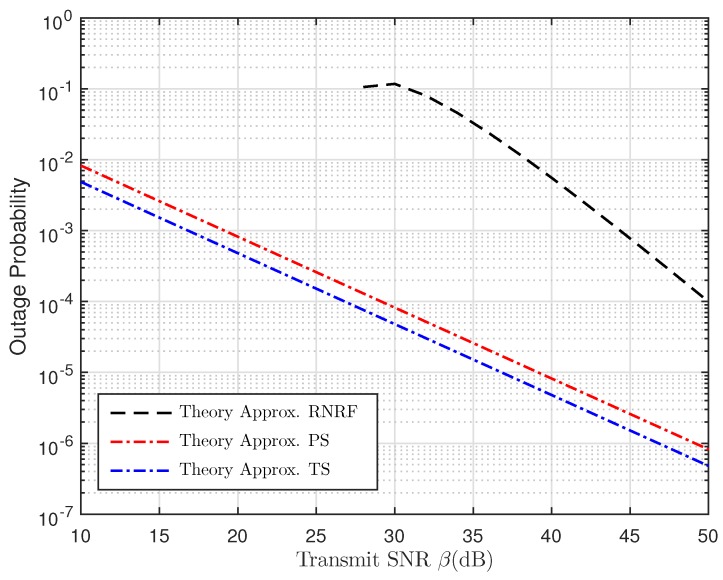
Comparisons between TSR WSN-NOMA, PSR WSN-NOMA, and random near NOMA user and random far NOMA user (RNRF) selection for the far users [30] in terms of OP versus the transmit SNR.

Figure 8 compares the OP of TSR WSN-NOMA, PSR WSN-NOMA, and random near NOMA user and random far NOMA user (RNRF) selection for the far users as a function of β (dB) corresponding to the analytical approximation results obtained in Equations (22), (38) and ([30], Equation (23)), respectively. It is obvious that the OP of TSR WSN-NOMA and PSR WSN-NOMA outperform that of RNRF selection for a large transmit SNR range because only R transmits the decoded symbol to B in the second time slot with complex power allocation at A to distinguish the power allocation coefficients ${\left|\rho_{i1}\right|}^{2}$ and ${\left|\rho_{i2}\right|}^{2}$. Meanwhile, in TSR WSN-NOMA and PSR WSN-NOMA, IT from A to B fully exploits the NOMA principle for further performance enhancement.

**Figure 9:**
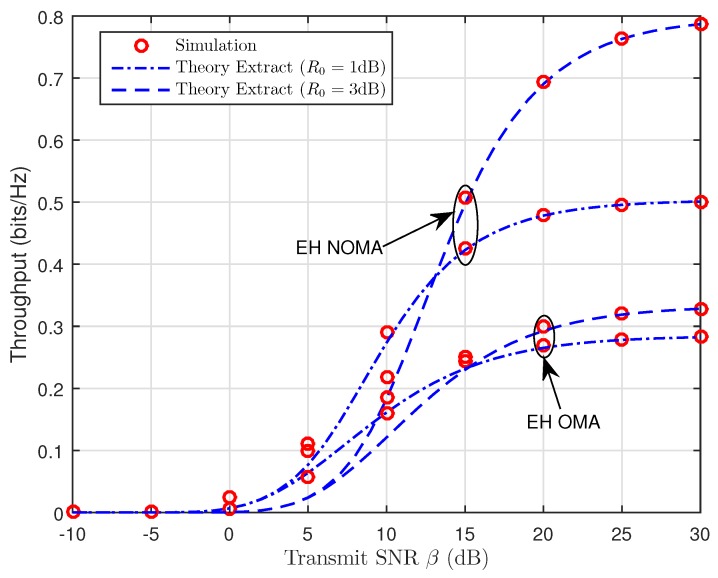
Throughput versus the transmit SNR (TSR WSN-NOMA).

**Figure 10:**
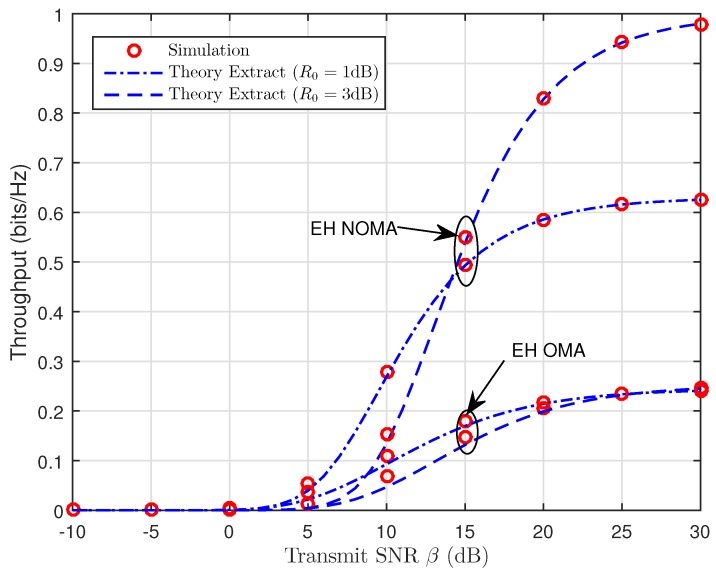
Throughput versus the transmit SNR (PSR WSN-NOMA).

Figure 9 and Figure 10 depict the throughput as a function of the transmit SNR for TSR WSN-NOMA and PSR WSN-NOMA protocols in delay-limited transmission mode for different values of the source transmission rate, $R_{0}$. We can see that throughput increases as the transmit SNR increases because the impact of $R_{0}=1$ (dB) is better than that of $R_{0}=3$ (dB). Besides that, we can find that our considered system enjoys better system performance over traditional OMA relaying systems since SIC is deployed at B, and it allows the transmission of $x_{2}$ in the second phase. With the help of R, the fading gain of $x_{1}$ is also improved with larger β. Additionally, it is noted that the dependence of throughput performance on $R_{0}$ (See in Equation (26) for TSR WSN-NOMA and Equation (40) for PSR WSN-NOMA, respectively) at relatively low transmission rates degrades the overall performance gains. On the other hand, in case of larger transmission rates $R_{0}$, R cannot decode much data correctly in a short amount of time.

**Figure 11:**
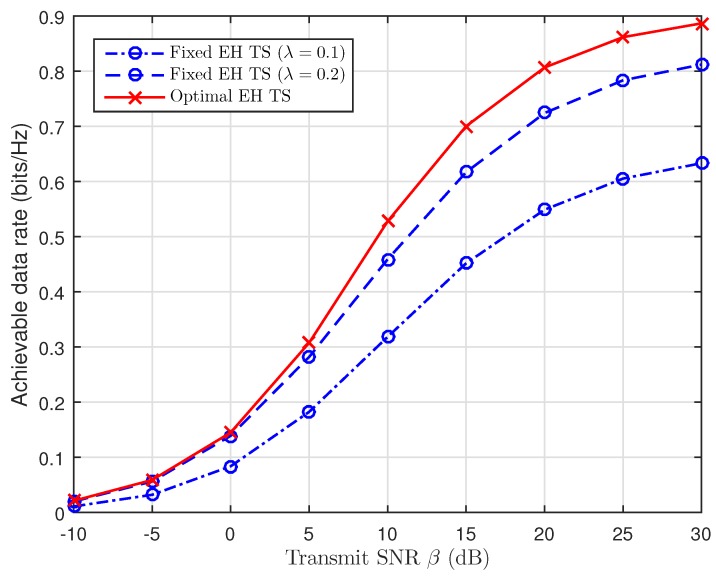
Achievable rate versus the transmit SNR with fixed values and the optimal EH TS.

In Figure 11, the achievable data rate at B with optimal values of TS is better than that of fixed TS ratio (λ=0.1,λ=0.2), because the values of λ are smaller than that of the optimal λ, and there is less time for EH. As a result, there is less energy harvested, and the throughput at B is poor due to the larger OP. The values of λ are greater than that of the optimal λ, because there is more time used for EH while IT process only receives a little time.

**Figure 12:**
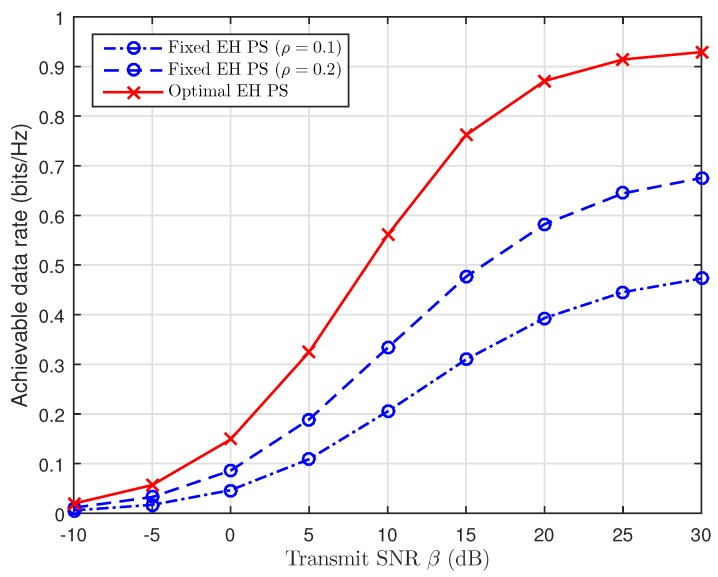
Achievable rate versus the transmit SNR with fixed values and the optimal EH PS.

Likewise, in Figure 12, the optimal values of PS ratio are observed to be better than that of the fixed PS ratio. Since the values of ρ are smaller than that of the optimal ρ, there is less power available for EH. Consequently, the values of ρ are greater than that of the optimal ρ, so there is more power consumed for EH, and less power is available for IT between A and the fixed DF scheme at R.

**Figure 13:**
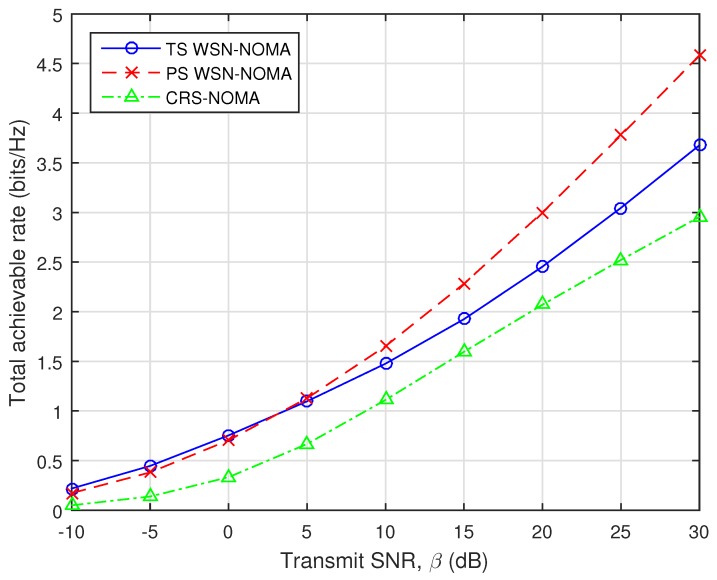
Total achievable rate against the transmit SNR.

The total achievable data rate with fixed DF relaying scheme versus the transmit SNR is illustrated in Figure 13. In this simulation, when the distances of all links are represented by the average power, we fix the distance parameters of the A–R, R–B, and A–B links as $\Omega_{X}$ = 10, $\Omega_{Y}$ = 2, and $\Omega_{Z}$ = 1 (dB), respectively. Furthermore, it can be seen that we give a fair comparison with an existing cooperative relaying system using NOMA (CRS-NOMA) in [17] by using the original simulation parameters. It can be observed that our considered system achieves better performance gains compared to CRS-NOMA. This is because, thanks to NOMA principle, $x_{1}$ and $x_{2}$ can be decoded by using SIC technique.

**Figure 14:**
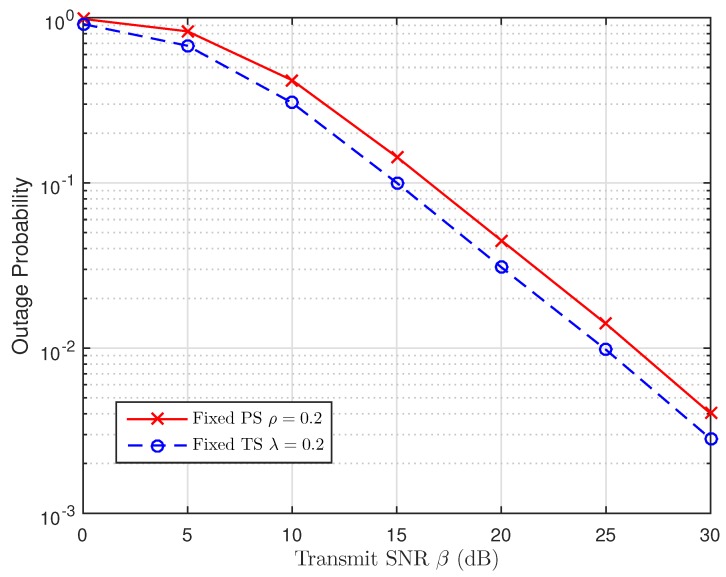
Comparison of TSR WSN-NOMA and PSR WSN-NOMA (Case 1).

**Figure 15:**
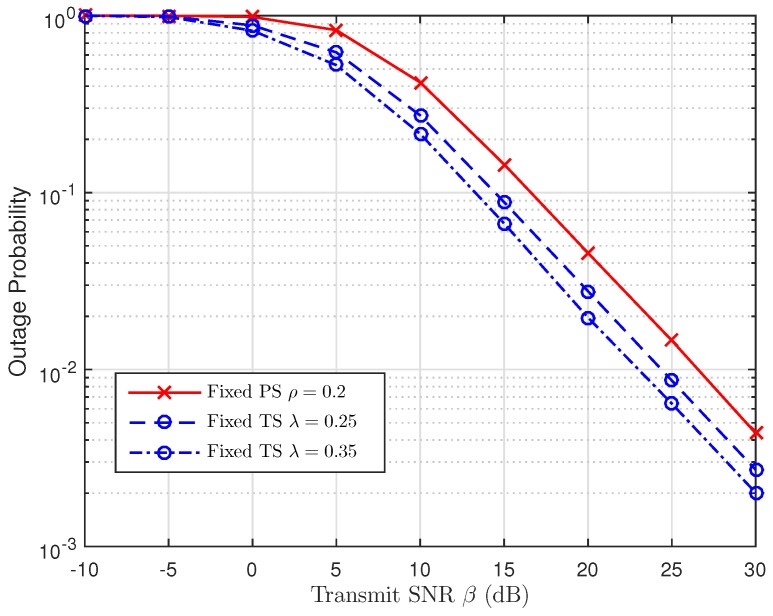
Comparison of TSR WSN-NOMA and PSR WSN-NOMA (Case 2).

**Figure 16:**
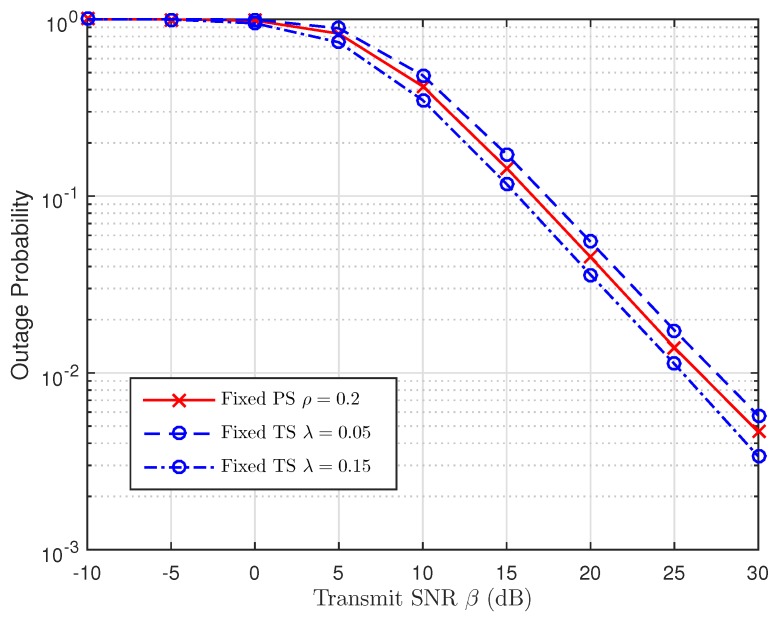
Comparison of TSR WSN-NOMA and PSR WSN-NOMA (Case 3).

As discussed in Section 5, we are going to use the simulation results to examine the impact of TS and PS ratios in Figure 14, Figure 15 and Figure 16, respectively. For this perspective, the system outage performance of PSR WSN-NOMA is higher than that of TSR WSN-NOMA because the system outage performance of TSR WSN-NOMA and PSR WSN-NOMA rely on the information received and energy harvested by R in the same channel conditions. In addition, to well maintain the balance between IT and EH in TSR WSN-NOMA, except the system transmission time assignment, information decoding and EH which are processed by TS receiver are sequenced over a transmission phase with different TS ratios. However, regarding PSR WSN-NOMA, except the system transmission time assignment and the power allocation, information decoding and EH are processed at the same time at R over a transmission phase with different PS ratios. For that reason, PS is proved to be more intelligent compared to TS in terms of the resource allocation.

## 7. Conclusions

In this work, an EH wireless sensor network using NOMA with TSR WSN-NOMA and PSR WSN-NOMA was considered. We presented our system performance analysis by obtaining closed-form expressions for the exact and approximate OP in both protocols, and the delay-limited throughput was also given. In addition, we provide theoretical comparisons between the two protocols, and the optimization problems for them were also solved to reduce OP and maximize the system data rate. Thanks to the simulation results, the robustness of the system was proven. Both TSR WSN-NOMA and PSR WSN-NOMA help the system achieve high-performance gain thanks to the optimal values of TS and PS ratios. The optimal TSR WSN-NOMA is better than the optimal PSR WSN-NOMA in terms of OP.

## Figures and Tables

**Table 1:** Important notations.

Symbols	Meanings
$P_{A}$	The transmit power of A.
$P_{R}$	The transmit power of R.
η	The energy efficiency, $\eta \in \left\{0,1\right\}$.
λ	The TS ratio of the EH receiver.
ρ	The PS ratio of the EH receiver.
$d_{X},d_{Y},d_{Z}$	The distances from A to R, R to B, and A to B, respectively.
${\left|X\right|}^{2}$, ${\left|Y\right|}^{2}$, ${\left|Z\right|}^{2}$	The channel gain RVs for the links from A to R, R to B, and A to B, respectively.
$\Omega_{X}$, $\Omega_{Y}$, $\Omega_{Z}$	The exponential parameters corresponding to ${\left|X\right|}^{2}$, ${\left|Y\right|}^{2}$,${\left|Z\right|}^{2}$, respectively.
$n_{0}$	The additive white Gaussian noise (AWGN) with mean power, $N_{0}$.
$\gamma_{0}$	The SNR threshold.
OP	The outage probability.
$F_{X},f_{X}$	The CDF/the PDF.
$E\left\{\left|.\right|\right\}$	The expectation operator.
$\mathrm{Pr}\left\{.\right\}$	The probability distribution function.
$K_{n}\left\{.\right\}$	The *n* order modified Bessel function of the second kind with the last equality.
W(x)	The Lambert *W* function W(x) is a set of solutions of the equation $x=W\left(x\right)e^{W(x)}$.
$W_{\mu ,v}\left(x\right)$	The Whittaker function.

**Table 2:** For TSR WSN-NOMA versus PSR WSN-NOMA.

Items	TSR WSN-NOMA	PSR WSN-NOMA
$P_{R,TS/PS}$	$2\eta \lambda {\left(1-\lambda \right)}^{-1}P_{A}{\left|X\right|}^{2}d_{X}^{-m}$	$\eta \rho P_{A}{\left|X\right|}^{2}d_{X}^{-m}$
$OP_{TS/PS}^{\left(x_{1}\right)}$	$1-e^{-\varepsilon_{1}}-e^{-\varepsilon_{2a/2b}}\left(1-e^{-\varepsilon_{1}}\right)\int_{z=0}^{\infty }\frac{1}{\Omega_{Z}}e^{-\frac{z}{\Omega_{Z}}}\Theta_{TS/PS}K_{1}\left(\Theta_{TS/PS}\right)dz$	
$OP_{TS/PS,\infty }^{\left(x_{1}\right)}$	$\varepsilon_{1}-\varepsilon_{1}\left(1-\varepsilon_{2a/2b}\right)e^{\frac{1}{2}\Theta_{TS/PS,\infty }}W_{-1,\frac{1}{2}}\left(\Theta_{TS/PS,\infty }\right)$	
$OP_{TS/PS}^{\left(x_{2}\right)}$	$1-e^{-\varepsilon_{1}}\int_{z=0}^{\infty }\frac{1}{\Omega_{Z}}e^{-\frac{z}{\Omega_{Z}}}\Theta_{TS/PS}K_{1}\left(\Theta_{TS/PS}\right)dz$	
$OP_{TS/PS,\infty }^{\left(x_{2}\right)}$	$1-\left(1-\varepsilon_{1}\right)e^{\frac{1}{2}\Theta_{TS/PS,\infty }}W_{-1,\frac{1}{2}}\left(\Theta_{TS/PS,\infty }\right)$	
Constants	$\varepsilon_{1}=\frac{d_{Z}^{m}\gamma_{0}}{\beta \Omega_{Z}},$	
	$\varepsilon_{2a}=\frac{d_{X}^{m}\gamma_{0}}{\beta \Omega_{X}},$	$\varepsilon_{2b}=\frac{d_{X}^{m}\gamma_{0}}{\beta \left(1-\rho \right)\Omega_{X}},$
	$\Theta_{TS}=\sqrt{\frac{2d_{X}^{m}d_{Y}^{m}\gamma_{0}}{\eta \lambda {\left(1-\lambda \right)}^{-1}\beta \Omega_{X}\Omega_{Y}}\left(\beta d_{Z}^{-m}z+1\right)},$	$\Theta_{PS}=\sqrt{\frac{4d_{X}^{m}d_{Y}^{m}\gamma_{0}}{\eta \rho \beta \Omega_{X}\Omega_{Y}}\left(\beta d_{Z}^{-m}z+1\right)},$
	$\Phi_{TS,\infty }=\frac{d_{X}^{m}d_{Y}^{m}d_{Z}^{-m}\Omega_{Z}\gamma_{0}}{2\eta \lambda {\left(1-\lambda \right)}^{-1}\Omega_{X}\Omega_{Y}}.$	$\Phi_{PS,\infty }=\frac{d_{X}^{m}d_{Y}^{m}d_{Z}^{-m}\Omega_{Z}\gamma_{0}}{\eta \rho \Omega_{X}\Omega_{Y}}.$

## Abbreviations

The following abbreviations are used in this manuscript:

NOMA	Non-orthogonal multiple access
SWIPT	Simultaneous wireless information and power transfer
WSN	wireless sensor network
SIC	Successive interference cancellation
TSR WSN-NOMA	Time-switching-based relaying WSN-NOMA
PSR WSN-NOMA	power splitting-based relaying WSN-NOMA
OP	Outage probability
